# School-Based Intervention as a Component of a Comprehensive Community Program for Overweight and Obesity Prevention, Sousse, Tunisia, 2009–2014

**DOI:** 10.5888/pcd12.140518

**Published:** 2015-09-24

**Authors:** Jihene Maatoug, Zineb Msakni, Nawel Zammit, Sana Bhiri, Imed Harrabi, Lamia Boughammoura, Slim Slama, Chaieb Larbi, Hassen Ghannem

**Affiliations:** Author Affiliations: Zineb Msakni, Nawel Zammit, Sana Bhiri, Imed Harrabi, Lamia Boughammoura, Chaieb Larbi, Hassen Ghannem, University Hospital Farhat Hached, Sousse, Tunisia; Slim Slama, Medical Officer, World Health Organization, Eastern Mediterranean Regional Office, Cairo, Egypt.

## Abstract

**Introduction:**

Combating obesity at an early age, by improving physical activity and nutrition-related behaviors, is vital to the prevention of more critical health concerns in adulthood. This intervention study evaluated the effectiveness of a school-based component of a community behavioral intervention on overweight and obesity rates of adolescents in Sousse, Tunisia.

**Methods:**

A quasi-experimental school-based intervention was conducted with an intervention group (in Sousse Jawhara and Sousse Riadh) and a control group (in Sousse Msaken). The intervention (which was a physical activity and nutrition program) lasted 3 years, with data at preintervention collected during the 2009–2010 school year and at postintervention collected during the 2013–2014 school year. Descriptive statistics and multivariate analysis were used to determine the effect of the intervention on risk of excess weight.

**Results:**

Results showed a significant increase in fruit and vegetable intake by the intervention group (*P* = .04). The intervention group had an increase in students in the normal weight category (*P* = .03) and a decrease in students in the overweight category (*P* = .03).The intervention effect was a protective factor against excess weight for the participating schoolchildren (OR, 0.84; *P* = .02).

**Conclusion:**

This study showed that a school-based intervention is successful in increasing healthy dietary habits and in reducing risk of excess weight. It also showed the importance of a multisectoral approach to provide an environment conducive to healthy behaviors for adolescents.

## Introduction

Overweight and obesity is a growing worldwide epidemic, cited by the World Health Organization as one of the leading public health challenges of the 21st century (1). Overweight and obesity are major risk factors for numerous chronic diseases including diabetes, cardiovascular disease, and cancer; these diseases result in the deaths of about 3.4 million adults each year (2). In particular, overweight and obesity in children and adolescents is significant because of the amount of data citing these conditions as predictors for adult obesity (3).

Once considered the concern of high-income countries, low-income and middle-income countries are quickly beginning to experience the same trends (4). It has also been predicted that the increase in adolescent obesity in the coming years will be 30% higher in low-income and middle-income countries than in the high-income countries (2). Tunisia in particular has seen a rise in overweight and obesity rates in adolescents as a result of the ongoing epidemiologic transition in the country (5). Changes in nutritional habits and improvements in health care delivery have rapidly changed health trends in the country, switching the country’s most common adverse health conditions from infectious diseases to noncommunicable diseases and their risk factors.

To properly combat the rise of overweight and obesity rates, risky behaviors must be modified. In determining these behaviors, many factors (including environmental and genetic factors) must be considered when assessing the cause of excessive weight gain. Despite the complexity of the underlying factors behind overweight and obesity, it is accepted that excess weight results from an imbalance between energy intake and energy expenditure (6). Understanding this imbalance has allowed numerous interventions to observe the importance of nutrition and physical activity in combating the obesity problem in adolescents (1).

Although a significant number of school-based studies look into modifying nutrition and physical activity behaviors to combat the obesity problem in developed countries, only a handful have been conducted in the developing world. In Tunisia, a school-based study on nutrition promotion is the only other known study on the topic (7).

Our study aimed to evaluate the effectiveness of “Schools in Health,” a school-based physical activity and nutritional behavior intervention, in reducing overweight and obesity rates among the schoolchildren of Sousse, Tunisia.

## Methods

The region of Sousse, covering an area of 2,612 km^2^, is composed of 15 delegations with 655,900 inhabitants estimated in 2013. Food expenditure is the highest expenditure (34.8% of the total). Adolescents are the main group that is moving away from traditional foods to a modern diet, linked to urbanization and increased economic level, which features increased consumption of white bread, dairy products, sugars, added fats, and fruits and decreased consumption of oils, grains, legumes, and vegetables (5).

### Study design

A school-based intervention (a physical activity and nutrition program) was conducted in Sousse using a quasi-experimental design with 2 groups: an intervention group comprised of all middle schools in the regions of Jawhara and Riadh, which have 62,663 and 65,333 inhabitants, respectively, and a control group that included all middle schools in the delegation of Msaken, which has 85,380 inhabitants. The distance between intervention and control groups is approximately 14 km. The data for this study were collected both preintervention and postintervention. Preintervention assessment was done during the 2009–2010 school year and postintervention assessment was completed during the 2013–2014 school year. The intervention lasted 3 years with a behavior modification program conducted for the intervention group, and no behavior modification program (usual intervention) for the control group (Figure).

**Figure:**
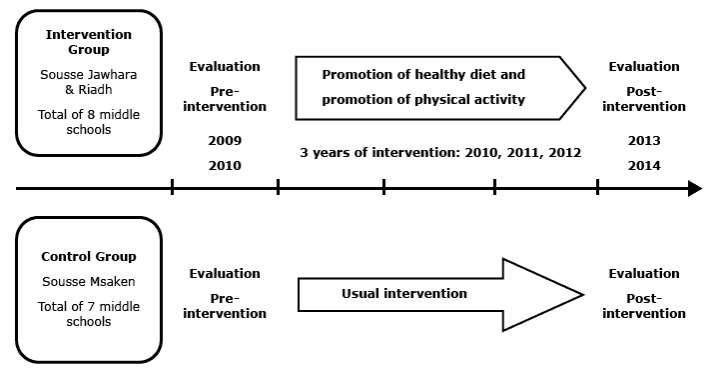
Timeline of quasi-experimental study (preintervention and postintervention assessment with control group) of an intervention program that is a component of a comprehensive community program for overweight and obesity prevention, Sousse, Tunisia, 2009–2014.

### Study population

The study population was composed of schoolchildren aged 11 to 16. The students in the intervention group were enrolled in the seventh and ninth grades of 8 middle schools and students in the control group were enrolled in 7 middle schools. Data were collected from a random selection of schoolchildren from all of the middle schools in the regions of both the intervention and control groups.

Sample size calculation for the different settings was based on a significance level of α = .05, power of test β = 20%, 2-sided test of hypothesis, and a 6% change in risk factor levels (unhealthy diet and physical inactivity), giving a total sample of 4,003 schoolchildren (1,929 intervention children and 2,074 control children).

The preintervention and postintervention assessments were gathered from a random (proportional and stratified) sampling of the current seventh and ninth graders in the schools. This study did not use a cohort design; however, changes observed in newly enrolled schoolchildren may be seen as a result of exposure to the larger community-based intervention that targeted adults and children through similar workplace and neighborhood programs. The school-based program highlighted in this study was a component of the community-based intervention.

### Data collection

A standardized, pretested questionnaire in Arabic was used to evaluate the physical activity behaviors and fruit and vegetable consumption of schoolchildren. Biometric measurements including height and weight of the schoolchildren were also collected. Body weight was recorded to the nearest 0.1 kg by using a portable electronic scale (PS 07, Beurer GmbH) taken with participants wearing a light layer of clothing. Standing height was measured with the participants standing barefoot and was recorded to the nearest 0.5 cm. The questionnaire was self-administered in schools in the presence of trained medical doctors to standardize and assist the schoolchildren in filling out the questionnaires and to define terminology. The main group of study investigators was the same at preintervention and postintervention assessment, but some interviewers left and others came during the course of the study. All interviewers, however, received the same training from the same trainers.

### Variable definitions

To define overweight and obesity among schoolchildren, we used the international cut-off values of body mass index according to age and sex (8).

Physical activity was assessed by using a combination of 2 questions: In a usual week, on how many days are you physically active for a total of at least 30 minutes per day?; and On these days that you are physically active for 30 minutes or more, for how long on average are you physically active?

The recommended level of physical activity for children (60 minutes of physical activity for 3 or more days per week) was established according to the definitions by the World Health Organization (9). Sedentary behavior was defined as any seated activity for more than 2 hours and included screen time per day; fast-food intake was defined as at least once in the previous week.

Fruit and vegetable consumption was assessed by using a combination of 2 questions: How many days per week do you usually eat fruits and vegetables?; and On the days you eat fruits and/or vegetables, on average how many servings of fruits and/or vegetables do you eat? We defined the recommended amount of fruits and vegetables as 5 or more servings per day.

### Intervention program

The 3-year school-based intervention program had 2 main components: educational lessons and environmental change. In terms of education, the program was heavily based on creating leader groups among the schoolchildren. Schoolchildren who were considered sources of influence for their peers were recommended by their teachers to participate in the intervention as “student leaders.”

The schoolchildren leaders were trained on how to motivate their peers to adopt healthy behaviors such at eating at least 5 fruits or vegetables daily or doing the recommended level of physical activity. They were given extra lessons on how to promote a positive image of healthy eating and physical activity among their peers and on how to advise their classmates properly. The educational sessions consisted of PowerPoint (Microsoft, Inc) presentations and interactive question-and-answer sessions on proper dietary habits and specifically how many portions of fruit and vegetables are recommended for daily consumption. The benefits of physical activity were also taught.

Schoolchildren leaders with the help of the project team organized educational events that consisted of open days for all schoolchildren, for parents, and for teachers. These events included presentation of posters, distribution of flyers, presentation of dances, and presentation of skits by children. These events were organized at least 3 times per school year in each school.

Education sessions in the classes were led by classroom teachers who were trained by the project team to teach students about healthy physical activity and diet habits. Content of classroom sessions consisted of interactive lessons. Teachers were asked to present at least 1 session to promote physical activity and 1 session to promote a healthy diet. These sessions presented the principles of healthy eating, the benefits of regular physical activity, and ways to incorporate physical activity into usual activities.

Student leader and teacher training consisted of workshops during 3 sessions of 1 day. Information on the benefits of healthy behaviors was followed up with tips on how to work the recommended physical activity and diet behaviors into the children’s daily routines. Free education programs on the management of obesity were also offered in the schools. To encourage physical activity, after-school soccer games were organized both within and between the schools. Fliers detailing the importance of healthy eating and physical activity habits were handed out to students and parents at twice throughout the program, and posters were hung in each school for the full 3 years.

Snack store clerks were encouraged to stock healthy alternatives to the usual sugary snacks. The alternatives consisted mostly of fresh fruits and vegetables or dairy products. Schoolchildren who chose the healthy snacks were rewarded with incentive stickers, which they collected for a prize at the end of each month.

### Data analyses

Statistical analyses were performed using SPSS 10.0 software (IBM Inc). Descriptive statistics (frequencies) and χ^2^ test were used to compare percentages, with .05 as the recognized level of significance. A multivariate analysis using a binary logistic regression was also conducted using normal weight versus excess weight (overweight, obese) as a dependent variable. The multivariate analysis was conducted separately for the intervention group and control group. The intervention effect is the change in the intervention group from before the intervention until the outcome after the intervention. Any changes in the control group were considered the result of usual activity without the intervention.

### Ethical consideration

Parents were asked for their informed consent before we recruited their children into the study. Parents had the choice to refuse their child’s participation. 

For ethical reasons, we conducted a short intervention for the control group after we collected postintervention data. This intervention consisted of open days to promote healthy eating and physical activity for the control children and their parents. We also provided the control children and their parents with information on managing overweight and obesity among schoolchildren.

## Results

The questionnaire was delivered to 4,003 schoolchildren for the collection of preintervention data, with a 94.6% response rate. Of those schoolchildren, 2,074 belonged to the control group and 1,929 belonged to the intervention group. In the postintervention examination, 4,275 (92.9% response rate) schoolchildren participated in study, with 2,106 of the participants belonging to the control group and 2,169 belonging to the intervention group. Boys accounted for 50.2% of the intervention group at preintervention assessment, and 48.7% of the participants at postintervention assessment. The 13-or-younger age group was 55.7% of the intervention group at preintervention and 54.7% at postintervention. For the control group, 46.5% of the schoolchildren at preintervention were boys and 47.7% were boys at postintervention. The 13-or-younger age group was 46.8% of the control group at preintervention and 52.7% at postintervention. There was no significant difference in sex or age between the preintervention and postintervention data of the intervention group (*P* = .35 and *P* = .53, respectively). In the intervention group, schoolchildren from a low socioeconomic background decreased from 24.7% at preintervention to 19% at postintervention, medium level decreased from 37.1% to 34.9% at postintervention, and high level increased from 38.2% to 46.1% postintervention. In the control group, children in the low socioeconomic level decreased from 24.4% at preintervention to 16.7% postintervention, medium level decreased from 43.1% to 38.1%, and high level increased from 32.5% to 45.2%. The socioeconomic levels of the intervention and control groups were significantly different from the levels of the preintervention and postintervention groups (*P* < .001).

The percentage of obese schoolchildren also decreased in the intervention group but not significantly while the percentage of obese schoolchildren in the control group increased significantly (*P* < .001) (Table 1).

**Table 1:** Weight Status of Schoolchildren Aged 11–16 Years by Sex and Age: Preintervention (2009–2010) and Postintervention (2013–2014) Data for Intervention Group and Control Group, Tunisia

Weight^a^/Sex/Age, y	Intervention			Control		
	Preintervention Assessment	Postintervention Assessment	*P* Value^b^	Preintervention Assessment	Postintervention Assessment	*P* Value^b^
**Normal weight, n (%)**	1,396 (72.4)	1,605 (75.5)	.03	1,658 (80.0)	1,566 (77.0)	.02
**Boys**	707 (73.1)	798 (77.2)	.04	772 (80.1)	756 (77.8)	.21
≤13	390 (70.3)	433 (75.7)	.04	339 (76.7)	410 (76.9)	.93
>13	317 (76.9)	365 (79.0)	.82	433 (83.0)	346 (78.8)	.10
**Girls**	689 (71.8)	807 (74.0)	.26	886 (79.9)	810 (76.2)	.04
≤13	365 (70.3)	427 (71.9)	.57	417 (79.0)	416 (75.2)	.14
>13	324 (73.5)	380 (76.5)	.29	469 (80.7)	394 (77.3)	.19
**Overweight, n (%)**	396 (20.6)	382 (18.0)	.03	321 (15.5)	328 (16.1)	.58
**Boys**	197 (20.4)	175 (16.9)	.04	145 (15.0)	142 (14.6)	.79
≤13	122 (22.0)	104 (18.2)	.11	77 (17.4)	79 (14.8)	.27
>13	75 (18.2)	71 (15.4)	.26	68 (13.0)	63 (14.4)	.55
**Girls**	199 (20.7)	207 (19.0)	.32	176 (15.9)	186 (17.5)	.30
≤13	102 (19.7)	119 (20.0)	.87	87 (16.5)	96 (17.4)	.70
>13	97 (22.0)	88 (17.7)	.10	89 (15.3)	90 (17.6)	.32
**Obese, n (%)**	135 (7.0)	138 (6.5)	.51	94 (4.5)	141 (6.9)	<.001
**Boys**	63 (6.5)	61 (5.9)	.57	47 (4.9)	74 (7.6)	.01
≤13	43 (7.7)	35 (6.1)	.28	26 (5.9)	44 (8.3)	.15
>13	20 (4.9)	26 (5.6)	.61	21 (4.0)	30 (6.8)	.05
**Girls**	72 (7.5)	77 (7.1)	.70	47 (4.2)	67 (6.3)	.03
≤13	52 (10.0)	48 (8.1)	.26	24 (4.5)	41 (7.4)	.04
>13	20 (4.5)	29 (5.8)	.37	23 (4.0)	26 (5.1)	.41

a The international cut-off values of body mass index according to age and sex were used (8).

b Pearson χ^2^ test used to determine *P* values.

Overall, the boys’ weight status improved postintervention more than the girls (Table 1). In the comparison between preintervention data and postintervention data, recommended fruit and vegetable intake improved significantly, from 30.0% to 33.2% postintervention (*P* = .03) while decreasing significantly in the control group from 40.2% to 35.0% (*P* = .001) (Table 2). Fried food and fast food intake also decreased in the intervention group but not significantly. There was a significant decrease (*P* < .001) in schoolchildren who walked or biked to and from school in the control group postintervention but a nonsignificant increase (from 72.2% to 74.0%) in the intervention group. There was a significant decrease (*P* = .01) in schoolchildren who did the recommended amount of physical activity in the intervention group, dropping from 29.1% to 25.5% postintervention. No significant changes were seen in sedentary behaviors in the intervention group while a significant decrease was observed on Sundays for the control group (*P* < .001).

**Table 2:** Behavior Assessment of Schoolchildren Aged 11–16 Years at Preintervention (2009–2010) and Postintervention (2013–2014): Data for Intervention Group and Control Group, Tunisia

Behavior	Intervention			Control		
	Preintervention Assessment	Postintervention Assessment	*P* Value^a^	Preintervention Assessment	Postintervention Assessment	*P* Value^a^
**Nutritional intake**						
Recommended amount of fruit and vegetables	565 (30.0)	702 (33.2)	.03	821 (40.2)	695 (35.0)	.001
Fried food (rarely or never)	143 (7.5)	169 (7.8)	.64	144 (7.0)	141 (7.0)	.99
Fast-food (never in past week)	571 (29.8)	609 (28.4)	.33	840 (40.6)	798 (39.3)	.43
**Physical activity**						
Recommended physical activity	554 (29.1)	536 (25.5)	.01	434 (21.1)	425 (21.2)	.89
Walk or bike to school	1,386 (72.2)	1,594 (74.0)	.19	1,645 (79.4)	1,442 (71.1)	<.001
**Sedentary behavior**						
On school day (>2 h)	734 (38.1)	869 (40.1)	.19	712 (34.3)	745 (35.4)	.48
On Sunday (>2 h)	1,237 (64.1)	1,411 (65.1)	.54	1,354 (65.3)	1,266 (60.1)	<.001

a Pearson χ^2^ test used to determine *P* values.

The multivariate analysis model, which used a binary logistic regression (Table 3), found a difference between the control group and the intervention group. Sex was included in the analysis for adjustment purposes. In the intervention group, protective factors against excess weight included the intervention effect (OR, 0.84; 95% CI, 0.73–0.97), increased age (OR, 0.91; 95% CI, 0.86–0.97), participation in recommended physical activity (OR, 0.80; 95% CI, 0.68–0.96), and recommended fruit and vegetable intake (OR, 0.84; 95% CI, 0.72–0.99). In contrast, the risk of excess weight increased with walking/biking to school (OR, 1.33; 95% CI, 1.11–1.56), and fast-food intake (OR, 1.18; 95% CI, 1.00–1.38). High socioeconomic level also increased the risk of excess of weight when compared with low socioeconomic level (OR, 1.25; 95% CI, 1.03–1.52).

**Table 3:** Multivariate Analysis Through Binary Logistic Regression of Risk for Excess Weight in Schoolchildren Aged 11–16 Years: Data for Intervention and Control Groups, Tunisia, 2009–2014

Variable	Intervention		Control	
	OR (95% CI)	*P* Value	OR (95% CI)	*P* Value
Intervention effect	0.84 (0.73–0.97)	.02	1.13 (0.97–1.32)	.12
Age	0.91 (0.86–0.97)	.003	0.94 (0.88–0.99)	.04
Sex (girls vs boys)	1.04 (0.89–1.21)	.63	1.07 (0.91–1.27)	.40
Socioeconomic level (medium vs low)	1.06 (0.86–1.29)	.60	1.17 (0.94–1.45)	.17
Socioeconomic level (high vs low)	1.25 (1.03–1.52)	.03	1.43 (1.14–1.78)	.002
Recommended physical activity	0.80 (0.67–0.96)	.02	1.06 (0.87–1.30)	.55
Walk or bike to school	1.33 (1.11–1.56)	.001	1.18 (0.98–1.42)	.07
Recommended fruit and vegetable intake	0.84 (0.72–0.99)	.04	0.80 (0.68–0.94)	.006
Fast-food intake (≥1 times in past week vs never)	1.18 (1.00–1.38)	.045	1.06 (0.91–1.25)	.44

Abbreviations: OR, odds ratio; CI, confidence interval.

## Discussion

The aim of this study was to evaluate the effectiveness of a physical activity and nutritional behavior intervention in reducing the overweight and obesity rates of schoolchildren of Sousse, Tunisia. This evaluation of the intervention showed promising progress toward a reduction in overweight and obesity rates in schoolchildren. Through the encouragement of positive lifestyle changes, schoolchildren in the intervention group were able to significantly improve their weight status, leading to a decrease in the overweight categories and an increase in the normal weight category.

The design of this study as a quasi-experimental school-based intervention with a reference group was one of its greatest strengths. The establishment of the program as a component of a larger community-based intervention limits our ability to categorize the intervention as strictly school-based. However, the design allowed for a comprehensive evaluation of the data, and in a developing country like Tunisia, it is a valuable stepping stone for future programs. Regions were selected based on proximity to the research center. Nonrandomization can lead to incomparability of the intervention and control groups. However, in data analysis, we compared only preintervention data with postintervention data and did not compare intervention group data with control group data.

We recognize that a quasi-experimental design cannot ensure that observed changes are the effect of the intervention. However, the intervention program was beneficial for schoolchildren.

The self-assessment questionnaire was another limitation of the program. Although the questionnaire was pretested and administered by professionals, false estimations of diet and physical activity behaviors by the schoolchildren are a possibility. This program also lacked effective environmental change. The occurrence of the Tunisian revolution in 2011 made it difficult for the program to enforce planned policy and infrastructural improvements as they were not considered a priority by schools and policy makers at the time. This program did, however, show the importance of such changes, and future interventions should consider including them in their programs. Findings from this study can be used to show the need for environmental modification, particularly in policy regulation that affects the surrounding the food environment and in decisions about infrastructure in the form of safe physical activity spaces.

Despite reducing the prevalence of overweight, little difference was observed in positive behavioral modifications among the schoolchildren. The most significant improvement was seen in fruit and vegetable intake. Other school-based interventions also encountered a lack of consistent behavioral results (10–12). One intervention in Australia showed inconsistent behavioral outcomes in dietary and physical activity behaviors despite the positive reduction in anthropometric measurements (11). In contrast, numerous other interventions showed positive changes in nutritional and physical activity behaviors but were unable to also show significant decreases in overweight and obesity (12,13). The most successful programs in reducing the prevalence of overweight and obesity were the ones that included some type of environmental change in the intervention (14). These changes included creating school-based gardens, making structural changes to physical activity courses, and making various policy and infrastructural changes (14). One intervention in Germany reduced the risk of overweight in schoolchildren by 31% at postintervention by installing water fountains in the intervention schools (8).

In terms of behavioral change, this intervention was able to show significant improvements in nutritional intake, similar to findings by other school-based studies (10,15). Findings from this study are also consistent in showing that schoolchildren who consumed more fast food increased their risk of overweight or obesity (16). One survey also presents evidence that children who live near fast-food restaurants have higher risks of overweight and obesity than children who do not (17). It may be interesting for future studies to take into consideration the number of fast-food establishments that schoolchildren pass on their way to school. This consideration is suggested because of the discovery that walking and biking to school seems to be raising the risk for excess weight in schoolchildren who participated in our intervention. The popular opinion remains, however, that frequent walking and biking is a beneficial way of maintaining a healthy weight (18).

Improvements in physical activity behaviors were not as significant. The lack of safe places such as parks and sport fields where adolescents can be active may have prevented the intervention group from having an increase in getting the recommended level of physical activity. Some interventions did not see a significant increase in physical activity (19,20), while others did (21,22). One school-based intervention conducted in Belgium was able to significantly increase physical activity behaviors by providing game equipment to schoolchildren during recess and lunch breaks (22). The intervention had the advantage of a small group of 249 schoolchildren, and as a result they were able to supply the students with accelerometers and enough game equipment to increase physical activity behaviors (22). Because this intervention program was large, we could not use measuring devices, and distributing enough game equipment to satisfy the number of participants was impossible.

One review of numerous school-based physical activity interventions noted that interventions that used classroom teachers instead of researcher staff showed more significant positive results (23). Another study showed that interventions with family-based components were more effective than school-based components alone in increasing physical activity behaviors of schoolchildren (24). Establishing a healthy environment is critical to the prevention of risky lifestyle behaviors by adolescent schoolchildren (25). Parents play a large role in modeling healthy behaviors and in promoting an environment that encourages healthy eating and physical activity behaviors (26). The discovery, by our study and others, that excess weight in low-income and middle-income income countries is a problem primarily seen in adolescents from high socioeconomic backgrounds, further increases the importance of the role of parents (27). However, as children begin spending more time at school, the responsibility of schools to encourage healthy behaviors becomes even more important (11). School lunch programs and physical activity courses make it easier for numerous schools to regulate the nutritional and physical activity habits of adolescents (14). Creating a healthy environment for children requires many sectors of the community to participate in implementing societal, infrastructural, and policy change.

## References

[1] World Health Organization. Childhood overweight and obesity. Geneva (CH): World Health Organization; 2014. http://www.who.int/dietphysicalactivity/childhood/en/. Accessed November 20, 2014.

[2] World Health Organization. Commission on Ending Childhood Obesity hold hearing with non-state actors. Geneva (CH): World Health Organization; 2014. http://www.who.int/end-childhood-obesity/en/. Accessed November 20, 2014.

[3] Whitaker RC , Wright JA , Pepe MS , Seidel KD , Dietz WH . Predicting obesity in young adulthood from childhood and parental obesity. N Engl J Med 1997;337(13):869–73. 10.1056/NEJM199709253371301 9302300

[4] World Health Organization. Obesity. Geneva (CH): World Health Organization; 2014. http://www.who.int/topics/obesity/en/. Accessed November 20, 2014.

[5] Aounallah-Skhiri H , Romdhane HB , Traissac P , Eymard-Duvernay S , Delpeuch F , Achour N , Nutritional status of Tunisian adolescents: associated gender, environmental and socio-economic factors. Public Health Nutr 2008;11(12):1306–17. 10.1017/S1368980008002693 18561866

[6] Spiegelman BM , Flier JS . Obesity and the regulation of energy balance. Cell 2001;104(4):531–43. 10.1016/S0092-8674(01)00240-9 11239410

[7] Kebaili R , Harrabi I , Maatoug J , Ghammam R , Slim S , Ghannem H . School-based intervention to promote healthy nutrition in Sousse, Tunisia. Int J Adolesc Med Health 2014;26(2):253–8. 10.1515/ijamh-2013-0306 24096439

[8] Cole TJ , Bellizzi MC , Flegal KM , Dietz WH . Establishing a standard definition for child overweight and obesity worldwide: international survey. BMJ 2000;320(7244):1240–3. 10.1136/bmj.320.7244.1240 10797032 PMC27365

[9] World Health Organization. Global recommendation on physical activity for health. Geneva (CH): World Health Organization; 2011. http://www.who.int/dietphysicalactivity/physical-activity-recommendations-5-17years.pdf?ua=1. Accessed November 20, 2014.

[10] Lehto R , Määttä S , Lehto E , Ray C , Te Velde S , Lien N , The PRO GREENS intervention in Finnish schoolchildren — the degree of implementation affects both mediators and the intake of fruits and vegetables. Br J Nutr 2014;112(7):1185–94. 10.1017/S0007114514001767 25106046

[11] Millar L , Kremer P , de Silva-Sanigorski A , McCabe MP , Mavoa H , Moodie M , Reduction in overweight and obesity from a 3-year community-based intervention in Australia: the ‘It’s Your Move!’ project. Obes Rev 2011;12(Suppl 2):20–8. 10.1111/j.1467-789X.2011.00904.x 22008556

[12] Caballero B , Clay T , Davis SM , Ethelbah B , Rock BH , Lohman T , Pathways Study Research Group. Pathways: a school-based, randomized controlled trial for the prevention of obesity in American Indian schoolchildren. Am J Clin Nutr 2003;78(5):1030–8. 14594792 10.1093/ajcn/78.5.1030 PMC4863237

[13] Austin SB , Kim J , Wiecha J , Troped PJ , Feldman HA , Peterson KE . School-based overweight preventive intervention lowers incidence of disordered weight-control behaviors in early adolescent girls. Arch Pediatr Adolesc Med 2007;161(9):865–9. 10.1001/archpedi.161.9.865 17768286

[14] Ickes MJ , McMullen J , Haider T , Sharma M . Global school-based childhood obesity interventions: a review. Int J Environ Res Public Health 2014;11(9):8940–61. 10.3390/ijerph110908940 25170684 PMC4198999

[15] Wind M , Bjelland M , Pérez-Rodrigo C , Te Velde SJ , Hildonen C , Bere E , Appreciation and implementation of a school-based intervention are associated with changes in fruit and vegetable intake in 10- to 13-year old schoolchildren — the Pro Children study. Health Educ Res 2008;23(6):997–1007. 10.1093/her/cym078 18156147

[16] Mohammadifard N , Sarrafzadegan N , Ghassemi GR , Nouri F , Pashmi R . Aliteration in unhealthy nutrition behaviors in adolescents through community intervention: Isfahan Healthy Heart Program. ARYA Atheroscler 2013;9(1):89–97. 23696765 PMC3653262

[17] Poti JM , Popkin BM . Trends in energy intake among US children by eating location and food source, 1977–2006. J Am Diet Assoc 2011;111(8):1156–64. 10.1016/j.jada.2011.05.007 21802561 PMC3148484

[18] Mendoza JA , Watson K , Nguyen N , Cerin E , Baranowski T , Nicklas TA . Active commuting to school and association with physical activity and adiposity among US youth. J Phys Act Health 2011;8(4):488–95. 21597121 10.1123/jpah.8.4.488 PMC3115568

[19] Colín-Ramírez E , Castillo-Martínez L , Orea-Tejeda A , Vergara-Castañeda A , Keirns-Davis C , Villa-Romero A . Outcomes of a school-based intervention (RESCATE) to improve physical activity patterns in Mexican children aged 8–10 years. Health Educ Res 2010;25(6):1042–9. 10.1093/her/cyq056 20884847

[20] Digelidis N , Papaioannou A , Laparidis K , Christodoulidis T . A one-year intervention in 7th grade physical education classes aiming to change motivational climate and attitudes towards exercise. Psychol Sport Exerc 2003;4(3):195–210. 10.1016/S1469-0292(02)00002-X

[21] Simon C , Wagner A , DiVita C , Rauscher E , Klein-Platat C , Arveiler D , Intervention centred on adolescents’ physical activity and sedentary behaviour (ICAPS): concept and 6-month results. Int J Obes Relat Metab Disord 2004;28(Suppl 3):S96–103. 10.1038/sj.ijo.0802812 15543228

[22] Verstraete SJ , Cardon GM , De Clercq DL , De Bourdeaudhuij IM . Increasing children’s physical activity levels during recess periods in elementary schools: the effects of providing game equipment. Eur J Public Health 2006;16(4):415–9. 10.1093/eurpub/ckl008 16431866

[23] Dobbins M , Husson H , DeCorby K , LaRocca RL . School-based physical activity programs for promoting physical activity and fitness in children and adolescents aged 6 to 18. Cochrane Database Syst Rev 2013;2:CD007651. 23450577 10.1002/14651858.CD007651.pub2 PMC7197501

[24] Salmon J , Booth ML , Phongsavan P , Murphy N , Timperio A . Promoting physical activity participation among children and adolescents. Epidemiol Rev 2007;29(1):144–59. 10.1093/epirev/mxm010 17556765

[25] Gao Y , Huang Y , Zhang Y , Liu F , Feng CX , Liu T , Evaluation of fast food behavior in pre-school children and parents following a one-year intervention with nutrition education. Int J Environ Res Public Health 2014;11(7):6780–90. 10.3390/ijerph110706780 24983391 PMC4113844

[26] Story M , Sherwood NE , Himes JH , Davis M , Jacobs DR Jr , Cartwright Y , An after-school obesity prevention program for African-American girls: the Minnesota GEMS pilot study. Ethn Dis 2003;13(1, Suppl 1):S54–64. 12713211

[27] Dinsa GD , Goryakin Y , Fumagalli E , Suhrcke M . Obesity and socioeconomic status in developing countries: a systematic review. Obes Rev 2012;13(11):1067–79. 10.1111/j.1467-789X.2012.01017.x 22764734 PMC3798095

